# DESIGNING TUTORIALS TO AUGMENT MOBILITY FOR OLDER ADULTS WITH COGNITIVE IMPAIRMENT

**DOI:** 10.1093/geroni/igad104.0319

**Published:** 2023-12-21

**Authors:** Neil Charness, Nicholas Gray, Dorota Kossowska-Kuhn, Hunui Na, Michael Pervratil

**Affiliations:** Florida State University, Tallahassee, Florida, United States; Safety National, St. Louis, Missouri, United States; Florida State University, Tallahassee, Florida, United States; Florida State University, Tallahassee, Florida, United States; Psychology Department, Tallahassee, Florida, United States

## Abstract

The Augmenting User Geocoordinates & Mobility with ENhanced Tutorials (AUGMENT) project aims to support aging adults experiencing navigation difficulties accompanying cognitive impairments due to stroke, mild cognitive impairment, and traumatic brain injury. We describe the process and principles for designing tutorials for Uber and Google Maps, two applications that can support improved mobility for those with navigation difficulties associated with cognitive impairment. Initially we examined predictors of self-reported navigation difficulty in a sample (n=300) of older adults aging normally. In the multiple regression, self-reported memory problems (beta = .35), severity of memory problems (beta=.18), and female gender (beta=.16) were the strongest predictors of wayfinding difficulty in addition to a standardized spatial orientation test (beta=.13). Finding that memory difficulties were strongly related to navigation difficulties even in normal aging implied that tutorials could not make strong demands on retention and helped define the queries for interviews in the needs assessment phase. Next, we interviewed aging adults with cognitive impairment and their care partners about navigation problems, focusing on difficulties using navigation devices and applications. We also presented screenshots of the two target apps and asked simple questions about actions that could be taken. We then designed prototype tutorials using principles such as directing attention to relevant display features by highlighting the features, minimizing extraneous workload, and providing spaced retrieval practice for interactions with the interface. We also describe the iterative design process being used to refine the tutorials and the planned summative test of tutorial efficacy.

